# EBUS-guided Iodine-125 seed implantation in lymph node as a novel therapy for MCAO: A case report

**DOI:** 10.1097/MD.0000000000041198

**Published:** 2025-01-17

**Authors:** Huiwen Qian, Jianqiang Jin, Changguo Wang, Yuting Wang, Juxiang Ge, Jianan Huang, Cheng Ji

**Affiliations:** a Department of Respiratory and Critical Care Medicine, The First Affiliated Hospital of Soochow University, Suzhou, China.

**Keywords:** EBUS, malignant central airway obstruction, radioactive ^125^I seed

## Abstract

**Rationale:**

We report here a case of using iodine-125 (^125^I) seed implantation via endobronchial ultrasound (EBUS) in the treatment of malignant central airway obstruction (MCAO) in a patient with lung adenocarcinoma.

**Patient concerns:**

The patient still experienced MCAO after conventional bronchoscopic interventional therapy.

**Diagnoses:**

The patient was diagnosed as lung adenocarcinoma stage IV (T4N2M1a).

**Interventions:**

She received implantation of radioactive ^125^I seeds in the lesion inside the right middle lobe bronchus and 12R lymph node via bronchoscopy and EBUS, in combination with chemotherapy and immunotherapy.

**Outcomes:**

The patient’s MCAO has been effectively controlled.

**Lessons:**

The success of this case suggests that EBUS-guided ^125^I seed implantation to lymph nodes is a safe and effective therapeutic option for MCAO.

## 1. Introduction

Although freezing, laser, microwave and other treatments are conventionally used to manage malignant central airway obstruction (MCAO), their effectiveness is often time-limited and the rate of reobstruction is high. Stent implantation, external irradiation and after-loading radiotherapy are also the commonly used methods to control MCAO. However, not all the patients were suitable to receive these treatments. Here we report, for the first time, that implanting Iodine-125 (^125^I) seeds in the draining metastatic lymph nodes above the lesions via endobronchial ultrasound (EBUS) to treat MCAO for a patient who were not suitable for stent placement and external radiation therapy.

## 2. Case report

**Figure 1.**
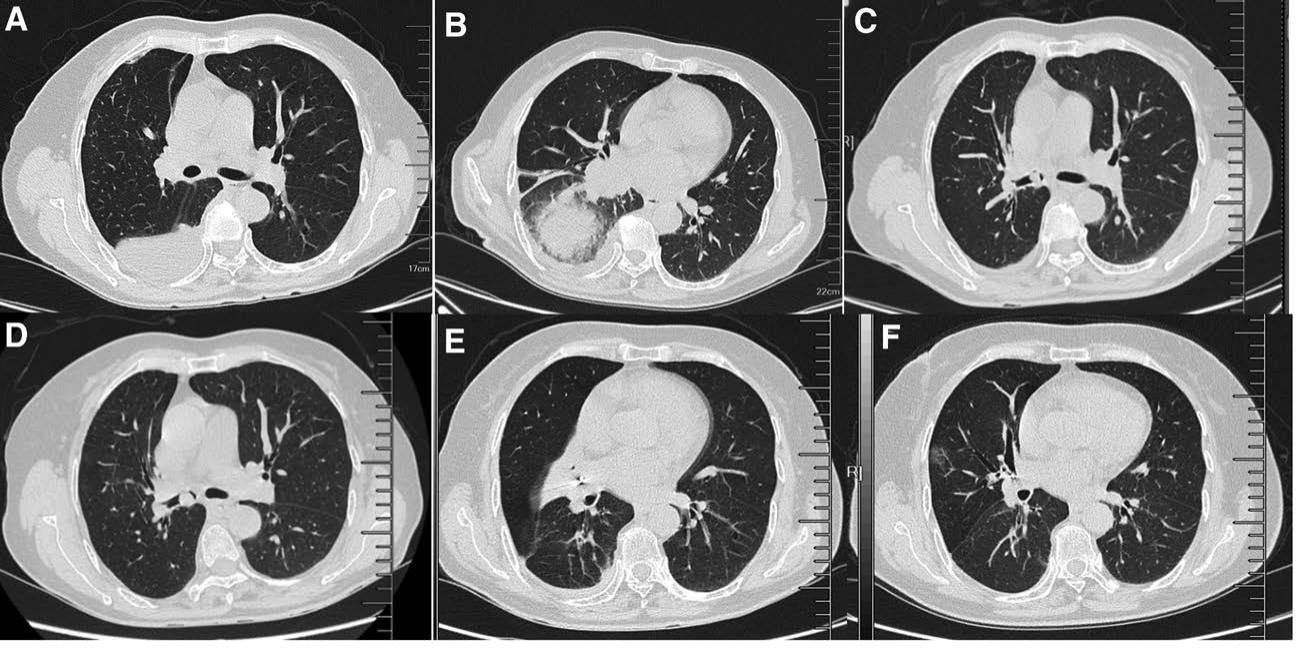
Chest CT of the patient. (A, B) CT: the presence of a right pulmonary mass, right lower lobar bronchus obstruction, and right pleural effusion. (C) CT: a new lesion appeared in the right intermediate bronchus. (D) CT: The lesion reobstruction in the right intermediate bronchus. (E) CT: ^125^I seeds were implanted in the lesion of the right middle lobe and 12R. (F) CT: lesion reduction and right middle lobe recruitment. CT = computed tomography.

A previously healthy 64-year-old female patient with a recurring cough and a large mass in right lower lung that showed right lower lung bronchial obstruction (Fig. 1A, B). The patient was diagnosed as lung adenocarcinoma stage IV (T4N2M1a) by bronchoscopy lung biopsy, with a *BRAF V600E* mutation and programmed death ligand 1 expression exceeding 50%. The Eastern Cooperative Oncology Group Performance Status of the patient was 1 score. Treatment consisting of targeted therapy with “Dabrafenib plus Trametinib,” along with “Pemetrexed” chemotherapy, was given to this patient. Partial response was observed in the patient 1 month later. However, the patient experienced fever associated to the use of targeted drugs, so the patient stopped targeted therapy and continued receiving chemotherapy with “Pemetrexed.”

**Figure 2.**
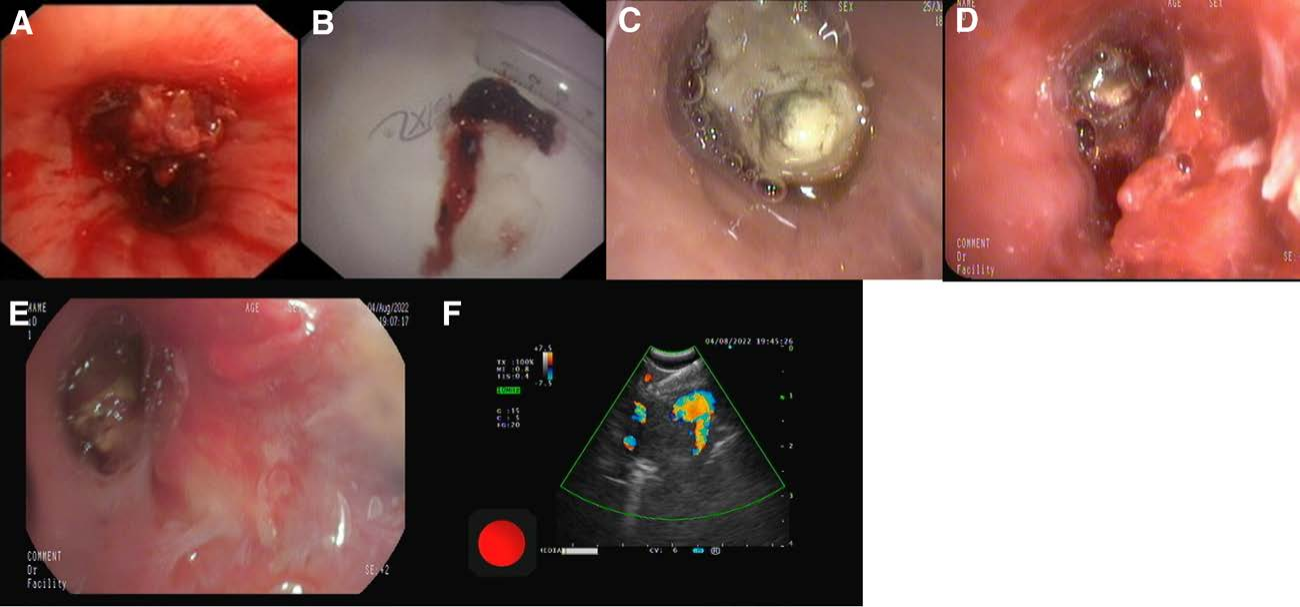
Bronchoscope and radioactive ^125^I seed implantation. (A, B) Bronchoscopy conducted on June 21, 2022: a new lesion in the right intermediate bronchus that had spread to the right middle lobe bronchus and the lesion was removed using snare. (C, D) Bronchoscopy on July 25, 2022: the right intermediate bronchus was entirely obstructed. Despite repeated use of snare to remove new lesion from the right middle bronchus, the distal segment remained invisible. (E, F) Implantation of radioactive ^125^I seeds in the right middle lobe lesion and 12R lymph node via bronchoscopy and EBUS guidance.

Subsequently, the patient complained chest tightness and wheezing. Chest CT revealed a new lesion in the right intermediate bronchus (Fig. 1C). Guided by bronchoscopy, we then used an electric snare to remove the new lesion (Fig. 2A, B). Considering the local progression of the tumor, the treatment was modified, which included chemotherapy (Pemetrexed plus Carboplatin) and immunotherapy (Sintilimab). Nevertheless, less than 1-month postdischarge, the patient experienced recurring MCAO (Fig. 1D). Bronchoscopy revealed that the right intermediate bronchus was entirely obstructed. Despite repeated use of the snare to remove new lesion from the right middle bronchus, the distal segment remained invisible (Fig. 2C, D).

Within merely a month, the patient experienced MCAO twice. In an effort to resolve this issue, the patient received bronchoscope-guided ^125^I seed implantation. Three ^125^I seeds with an activity of 0.65 mci were released in the lesion in the right middle lobe bronchus by the guide of conventional bronchoscopy, and meanwhile 4 ^125^I seeds with an activity of 0.65 mci were implanted at a 0.5 cm interval in the right group 12 (12R) enlarged lymph nodes located at the anterior to the opening of the right middle and lower lobe guided by EBUS (Fig. 2E, F). Subsequently, the patient received regular chemotherapy in combination with immunotherapy. Following these treatments, MCAO had never recurred and the lesion continued to shrink (Fig. 1E, F).

## 3. Discussion

The patient that we reported in this case experienced drug-associated fever subsequent to dual target therapy and local progression after discontinuing targeted drugs. Although we modified the systemic treatment, the recurrence of MCAO necessitated consideration of appropriate local treatment.

Radioactive ^125^I seed implantation is a form of brachytherapy that offers several advantages including short treatment distance, high central dose and continuous action, as compared to the conventional external irradiation. The application of ^125^I seed implantation is extensively employed in the local treatment of tumors. A study conducted on stage III NSCLC patients demonstrated that the combination of ^125^I seed implantation with chemotherapy yielded a 93.4% effective rate, with a median survival time of 24 months.^[1]^ The advancement of brachytherapy and respiratory interventional technology has made the radioactive ^125^I seed implantation as a viable treatment option for MCAO.^[2]^ Previous studies reported that both bronchoscopy-guided and CT-guided ^125^I seed implantation yielded favorable and safe outcomes.^[3,4]^ The introduction and clinical utilization of ^125^I seed stent has enriched the treatment of MCAO.^[5]^ In the current case, the tumor had spread from the distal end to the center, resulting in occlusion of the distal lumen, rendering stent implantation unsuitable. The patient had atelectasis in the middle lobe of the right lung, making it difficult to control the range and dosage for external irradiation. Furthermore, the catheter was unable to reach the distal end of the lesion, making after-loading radiotherapy unsuitable for the patient. As such, the employment of ^125^I seed implantation has demonstrated distinctive benefits in treatment of MCAO.

^125^I seeds are usually implanted in the lesion to control tumor growth; our team reported for the first time that implanting ^125^I seeds in the draining metastatic lymph nodes above the lesions through EBUS to managing the issue of MCAO that grows from distal to central. This approach not only alleviates the MCAO but also addresses the issue of implanted seed fixation in the body, while mitigating the radiation pollution that may result from seed expulsion.

**Figure 3.**
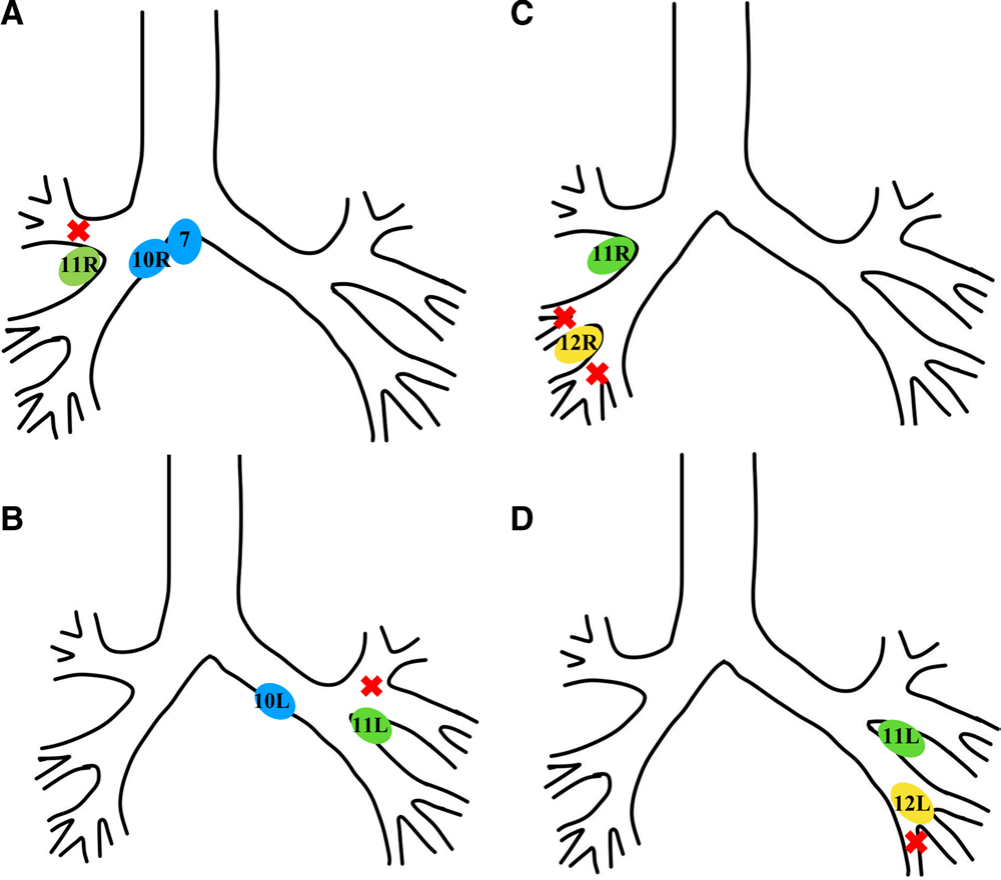
Proposed use of the implantation of ^125^I seeds into lymph nodes. (A, B) If the lesion is located in the upper lobe of the right (left) lung, ^125^I particles can be implanted through lymph node puncture in the 10th or 11th group of the right (left) lung. If the lesion in the upper lobe of the right lung involves the right main bronchus, the 7th group of lymph nodes can also be selected; (C, D) If the lesion is located in the middle/lower lobe of the right lung (lower lobe of left lung), ^125^I particles can be implanted through lymph node puncture in the 11th or 12th group of the right (left) lung.

Here we propose that the implantation of ^125^I seeds into positive lymph nodes may be considered for patients who satisfy the following criteria: the tumor exhibits growth from the distal to the proximal direction in the bronchus, resulting in airway stenosis; metastatic lymph nodes are present at the proximal end of the tumor; and the target lymph node possesses an appropriate EBUS puncture pathway (Fig. 3). The criteria for selecting the implantation site of ^125^I particles is the metastatic lymph nodes or lesions around the opening of the narrow bronchus. However, the selection of target lymph nodes or treatment plan should be tailored to the individual conditions.

The use of EBUS-guided ^125^I seed implantation in lymph nodes provides a novel, safe, effective, and accessible therapeutic option for patients with MCAO who are not suitable for stent placement and external radiation therapy.

## Author contributions

**Conceptualization:** Cheng Ji.

**Funding acquisition:** Cheng Ji.

**Methodology:** Jianqiang Jin.

**Software:** Huiwen Qian, Jianqiang Jin, Juxiang Ge.

**Supervision:** Jianan Huang.

**Writing – original draft:** Huiwen Qian, Yuting Wang.

**Writing – review & editing:** Changguo Wang, Cheng Ji.
